# Cilioretinal Arteries and Cilioretinal Veins in Eyes with Pathologic Myopia

**DOI:** 10.1038/s41598-019-38616-5

**Published:** 2019-02-21

**Authors:** Takashi Watanabe, Kaori Kasahara, Soh Futagami, Yuxin Fang, Ran Du, Muka Moriyama, Kengo Uramoto, Tae Yokoi, Yuka Onishi, Takeshi Yoshida, Koju Kamoi, Jost B. Jonas, Kyoko Ohno-Matsui

**Affiliations:** 1 Department of Ophthalmology and Visual Science, Tokyo Medical and Dental University, Tokyo, Japan; 2 Department of Ophthalmology, Tokyo Metropolitan Health and Medical Treatment Corporation Tama-Nanbu Chiiki Hospital, Tokyo, Japan; 3 Department of Ophthalmology, Nissan Tamagawa Hospital, Tokyo, Japan; 4 Department of Ophthalmology, Medical Faculty Mannheim of the Ruprecht-Karls-University Heidelberg, Seegartenklinik Heidelberg, Germany

## Abstract

We investigated the clinical characteristics of cilioretinal arteries (CAs) and cilioretinal veins (CVs) in eyes with pathologic myopia. Ninety-five eyes with pathologic myopia and CAs were studied. The retrobulbar vessels from which the CAs originated were identified by indocyanine green angiography (ICGA). The results showed that 114 CAs were identified in the 95 eyes. ICGA showed that 60% of the CAs branched directly off the short posterior ciliary arteries (SPCAs) and 40% originated from the Zinn-Haller arterial circle (ZHAC). The SPCA-derived CAs tended to be located superiorly and served a large retinal area whereas the ZHAC-associated CAs tended to be located temporally and served mainly the macular area. In 15% of the 95 eyes, the CVs were observed to run parallel to the CAs. The CVs exited the eye at the same point where the CAs entered the eye. This study showed that CAs in eyes with pathologic myopia can be divided into those that are SPCA-derived and tend to emerge in the superior optic disc sector, and those that are ZHAC-associated and usually emerge temporally. An elongating peripapillary scleral flange in eyes with progressive axial myopia may lead to a change of chorioretinal vascular system.

## Introduction

A cilioretinal artery (CA) can be a major element of the retinal vascular system and is found in 6% to 49.5% of the eyes in a general population^1–7^. CAs have been reported to arise most commonly from the short posterior ciliary arteries (SPCAs) and occasionally from choroidal vessels and serve mostly the macula area. A PubMed search using the keyword ‘cilioretinal artery’ on November 27, 2017 extracted 247 articles. Most of these reports described central retinal artery occlusions occurring in eyes with CAs or occlusion of CAs themselves. Collier *et al*.^1^ examined the relationship between CAs and refractive error, however, none of the studies described CAs specifically in eyes with pathologic myopia.

CAs have been reported to emerge most commonly at the temporal region of the optic disc. Liu *et al*.^2^ examined the prevalence of CAs in 5,000 eyes of 2,500 individuals aged 8 to 20 years and who participated in the Diabetic Eye Disease Study in Shenyang/China. On stereo fundus photographs CAs were present in 923 (18.5%) of the 5,000 eyes, with 85% of the CAs being located in the temporal region, 15% in the nasal area, and 7% in both the temporal or nasal region. Analyzing stereo fundus photographs of 2,000 eyes of 1,000 individuals, Justice and coworkers found one or more CAs in 32.1% of the eyes^3^. Of the 782 eyes with CAs, 88.2% were located in the temporal field.

There have been only few studies on cilioretinal veins (CVs), and most of them were focused on opticociliary shunt vessels developing after a partial occlusion of the central retinal vein or branch retinal vein when passing through the lamina cribrosa.

The anatomy of the optic nerve head differs markedly between non-highly myopic eyes and highly myopic eyes, in particular if additional pathological myopic changes are present. The optic nerve head in highly myopic eyes is characterized by a secondary macrodisc with an enlarged and shallow optic cup and the development and enlargement of Bruch’s membrane-free parapapillary gamma zone and delta zone^4,5^. In highly myopic eyes, the peripapillary scleral flange is elongated and thinned, the peripapillary cerebrospinal fluid space is extended, and the distance between the peripapillary arterial circle of Zinn Haller (ZHAC) and the optic disc border (defined by the peripapillary border tissue of Jacoby and Elschnig) is enlarged. Since the CAs and CVs run through the intrapapillary and parapapillary region, since these regions get markedly changed during the development of high axial myopia and since previous investigations did not study highly myopic eyes, we conducted this study to examine the CAs and CVs in eyes with pathologic myopia. Another reason to examine highly myopic eyes was that the intrascleral and retrobulbar vessels connected with the CAs and CVs can be visualized better in highly myopic eyes than in non-highly myopic eyes^6,7^.

## Results

**Figure 1:**
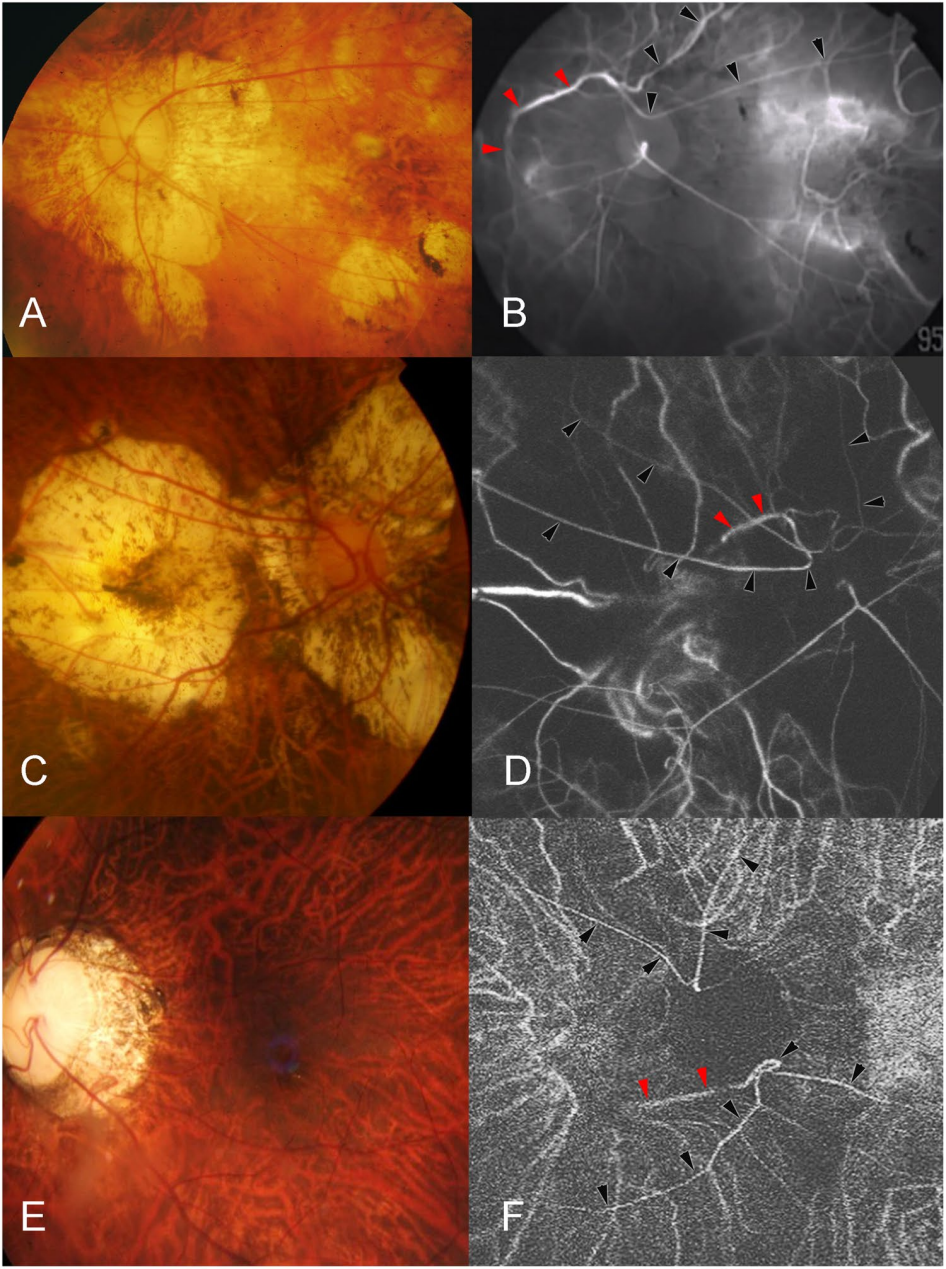
Cilioretinal arteries (CAs) deriving from the short posterior ciliary arteries (SPCAs). The left panel shows fundus photographs and the right panel shows the arterial phase of indocyanine green angiography (ICGA). **(A**,**B**) Left fundus photograph of the eye of a 70-year-old woman with an axial length of 31.1 mm. The ICGA image shows a large CA (black arrowheads) emerging at the upper pole of the optic disc from a SPCA. The central arterial trunk is located in the center of the optic disc and does not show any connection to the CA. The SPCA the CA is originating from is visualized (red arrowheads). **(C**,**D**) Right fundus photograph of the eye of a 75-year-old woman with an axial length of 28.8 mm. ICGA shows multiple CAs emerging from the upper sector of the optic disc (black arrowheads). The central arterial trunk is seen in the center of optic disc separated from the CAs. The SPCAs the CA originate from can be visualized (red arrowheads). **(E**,**F**) Left fundus of a 42-year-old woman with an axial length of 29.78 mm. The ICGA shows a CA emerging from the upper sector of the optic disc and another CA emerging from the lower sector of the optic disc (black arrowheads). The SPCA as origin of the CAs can be visualized on the ICGAs (red arrowheads).

**Figure 2:**
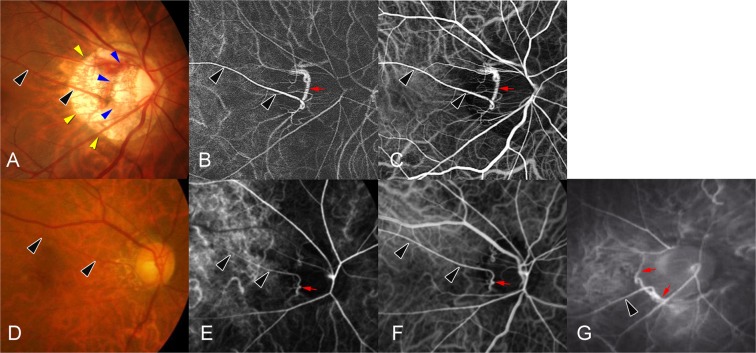
Cilioretinal arteries (CAs) deriving from the Zinn-Haller arterial circle (ZHAC). (**A**) Fundus photograph of a right highly myopic eye with an axial length of 28.1 mm. The CA (arrowheads) emerges from the border between parapapillary gamma zone (outlined by yellow arrowheads) and parapapillary delta zone (outlined by blue arrowheads) outside of the optic disc. (**B**) Arterial phase of the ICGA shows the ZHAC (arrow) in the temporal region and the CA (black arrowheads) derived from the ZHAC. (**C**) Venous phase of the ICGA. The CA (arrowheads) derived from the ZHAC (arrow) can visualized. (**D**) Right fundus of the eye of a 67-year-old woman with a temporal CA (arrowheads). (**E**) Arterial phase of the ICGA shows the ZHAC (arrow) and a CA (arrowheads) originating from the ZHAC. (**F**) Venous phase of ICGA. A CA (arrowheads) originating from the ZHAC (arrow) can visualized. (**G**) ICGA of the right fundus of the eye of a 29-year-old man. Axial length is 27.8 mm. A CA (arrowheads) deriving from the ZHAC (arrows) can be visualized outside of the optic disc.

**Figure 3:**
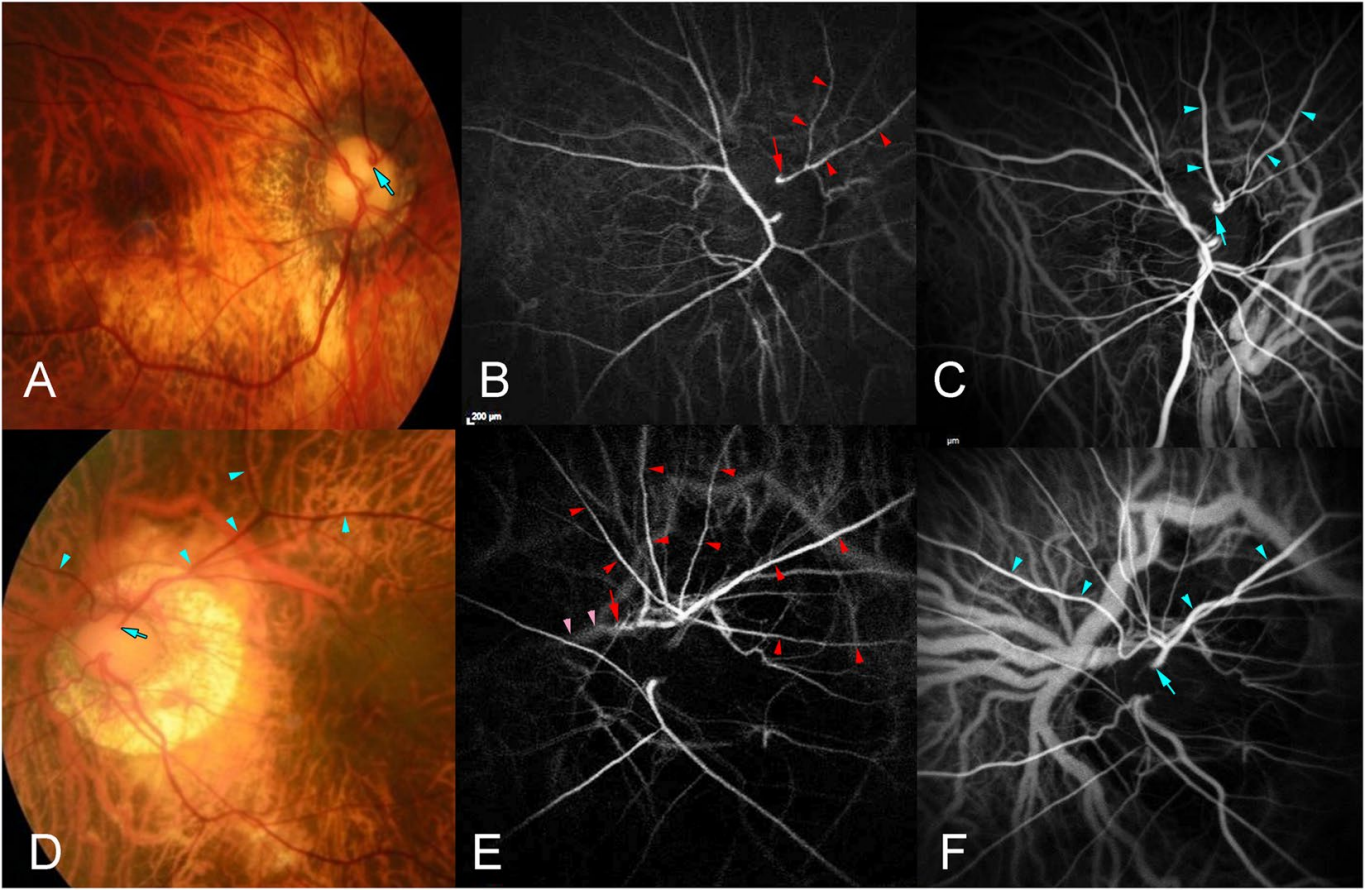
Co-existence of cilioretinal artery (CA) and cilioretinal vein (CV). (**A**) Right fundus of the eye a 60-year-old woman shows a CV (arrow) at the upper nasal border of the optic disc. (**B**) Arterial phase of indocyanine green angiogram (ICGA) shows the CA (arrowheads) emerging from the upper-temporal pole of the optic disc (arrow). (**C**) Venous phase of ICGA shows a CV (arrowheads) running parallel to the CA and which exits at the upper-temporal border of the optic disc. A posterior vortex vein with ampulla is also visualized inferior to the optic disc. (**D**) Left fundus of the eye a 43-year-old woman with a CV (arrowheads). The CV exits at the superior border of the optic disc (arrow). (**E**) Arterial phase of ICGA shows a large CA (red arrowheads) serving nearly one-half of the upper fundus. The CA (arrow) originates from a short posterior ciliary artery (pink arrowheads). (**F**) Venous phase of ICGA shows a large CV (arrowheads) running parallel to the CA. The CV exits at the upper border of the optic disc (arrow). A posterior vortex vein drains close to the superior optic disc border can also be detected.

The study included 95 highly myopic eyes of 86 patients with CAs (Figs 1–3). An agreement on the diagnosis of CAs and CVs was obtained for all cases between the two experienced examiners (TW, KOM). The mean age of the 86 patients was 57.2 ± 14.5 (range 24 to 84) years, and after excluding 36 pseudophakic eyes, the mean refractive error of the remaining 59 eyes was −14.2 ± 4.9 D with a range of −6.3 to −24.0 D. The mean axial length of the eyes was 30.2 ± 2.1 mm (range: 26.0–36.1 mm).

As a control, 1215 highly myopic eyes of 639 patients who had undergone indocyanine green angiography (ICGA) during the same examination period and who did not have any CAs as based on the examination of fundus photos, on fluorescein angiography and upon ICGA were extracted from our database. Overall, the prevalence of CAs among the population of our highly myopic patients was 7.3% (95 out of a total of 1310 eyes). Table 1 shows a comparison of clinical features between the patients with and without CAs. The results showed that highly myopic patients with CAs were significantly younger than those without CAs. The highly myopic eyes with CAs had significantly longer axial length, had significantly more frequently glaucoma, and had significantly more frequently category 4 of myopic maculopathy (=macular atrophy). There were no significant differences in the best corrected visual acuity (BCVA), refractive error, subfoveal choroidal thickness, the prevalence of lacquer cracks, and the prevalence of severe myopic maculopathy between the two groups (Table 1).

**Table 1:** Comparison of characteristics between the eyes with and without cilioretinal arteries (CAs).

	With CAs	Without CAs	*p-*value
Number of eyes	95	1215	
Age (years)*	57.2 ± 14.5	62.5 ± 13.6	0.001
Best corrected visual acuity (logMAR)*	0.35 ± 0.55	0.31 ± 0.46	0.53
Axial length (mm)*	30.2 ± 2.1	29.7 ± 2.2	0.03
Refractive error (μm)*	−14.2 ± 4.9	−13.2 ± 4.2	0.15
Subfoveal choroidal thickness (μm)*	61.8 ± 49.9	71.2 ± 54.6	0.15
Glaucoma^†^	41 (43.2%)	363 (29.9%)	0.007
Lacquer cracks^†^	34 (35.8%)	555 (45.7%)	0.06
Myopic choroidal neovascularization^†^	32 (33.7%)	357 (29.3%)	0.38
Category of myopic maculopathy			
Category 0^†^	1 (1.1%)	21 (1.7%)	0.62
Category 1^†^	17 (17.9%)	269 (22.1%)	0.34
Category 2^†^	29 (30.5%)	453 (37.3%)	0.19
Category 3^†^	23 (24.2%)	276 (22.7%)	0.74
Category 4^†^	25 (26.3%)	196 (16.1%)	0.01
Severe myopic maculopathy (≥category 2)^†^	77 (81.1%)	925 (76.1%)	0.28

*Unpaired t-test with Welch’s correction.

^†^Chi-square test.

### Location and original vessels of CAs

Eighty of the 95 eyes (84%) had only one CA, 12 eyes (13%) had two CAs, and 3 (3%) had 3 or more CAs. The total number of CAs in the 95 eyes was 114. The CAs were located most often in the superior sector (52 eyes; 46%) followed by the temporal sector (42 eyes; 37%), the nasal sector (11 eyes; 10%) and finally the inferior sector (9 eyes; 8%).

For 89 of the 114 CAs, the vessel of origin could be identified on the ICGA images, with 53 CAs (60%) branching directly from a SPCA (Fig. 1) and 36 CAs (40%) deriving from the ZHAC (Fig. 2). In the eyes of the remaining 25 CAs, intrascleral or retrobulbar blood vessels were not observed clearly even by ICGA, so that an identification of the vessels of origin of the CAs was difficult. All SPCA-derived CAs emerged within the optic disc (as defined by the peripapillary border tissue of Jacoby and Elschnig or the peripapillary ring), whereas 14 (39%) of the 36 ZHAC-associated CAs emerged within the disc area. The remaining 22 (61%) ZHAC-associated CAs emerged within the parapapillary region, usually from the border between parapapillary gamma zone and delta zone (Fig. 2).

Among the 53 SPCA-derived CAs, 38 CAs (72%) ran into the superior field, 8 CAs (15%) went into the temporal field, 4 CAs (8%) were directed into the inferior field, and 3 CAs (6%) went into the nasal field. CAs emerging from the superior optic disc edge as compared to CAs emerging from the inferior disc pole tended to cover a larger retinal area. In 8 eyes, the CAs covered nearly one half of the fundus (Supplementary Fig. 1).

Among the 36 ZHAC-associated CAs, 22 (61%) went into the temporal area, 10 (28%) into the superior area, 3 (8%) into the nasal area, and 1 (3%) into the inferior area.

In 14 of the 95 eyes (15%) with CAs, CVs ran parallel to CAs (Fig. 3). In these 14 eyes, the entry site of the CAs and the exit site of the CVs were located at the superior margin of the optic disc. None of the eyes with temporal CAs had a co-existing CV. In one of the 14 eyes, a posterior vortex vein penetrated the eye around the exit site of the CA and CV (bottom image, Fig. 3).

Table 2 shows a comparison between the 14 eyes with both CAs and CVs and the remaining 81 eyes with CAs but without CVs. The results revealed that the eyes with CAs and CVs had a significantly higher prevalence of glaucoma than those with CAs only (78.6% vs 37.0%, *P* = 0.0038, Chi square tests). In addition, the eyes with CAs and CVs had significantly less frequently lacquer cracks than the eyes with CAs only (7.1% vs 40.7%, *P* = 0.016, Chi square tests). There was no statistically significant difference (all *P* > 0.05) between both groups in age, refractive error, axial length, subfoveal choroidal thickness, prevalence of myopic choroidal neovascularization (CNV), and the prevalence of severe myopic maculopathy.

**Table 2:** Comparison of characteristics between the eyes with both cilioretinal arteries (CAs) and cilioretinal veins (CVs) and the eyes with CAs only.

	Both CAs and CVs	CAs only	*p-*value
Number of eyes	14	81	
Age (years)*	60.6 ± 15.6	56.6 ± 14.4	0.38
Axial length (mm)*	30.6 ± 2.1	30.2 ± 2.1	0.55
Refractive error (μm)*	−13.3 ± 4.7	−13.8 ± 5.1	0.82
Subfoveal choroidal thickness (μm)*	74.1 ± 58.4	59.26 ± 48.2	0.45
Glaucoma^†^	11 (78.6%)	30 (37.0%)	0.004
Lacquer cracks^†^	1 (7.1%)	33 (40.7%)	0.02
Myopic choroidal neovascularization^†^	5 (35.7%)	27 (33.3%)	0.86
Category of myopic maculopathy			
Category 0^†^	0 (0%)	1 (1.2%)	0.68
Category 1^†^	2 (14.3%)	15 (18.5%)	0.70
Category 2^†^	7 (50.0%)	22 (27.2%)	0.09
Category 3^†^	1 (7.1%)	22 (27.2%)	0.11
Category 4^†^	4 (28.6%)	21 (25.9%)	0.84
Severe myopic maculopathy (≥category 2)^†^	12 (85.7%)	65 (80.2%)	0.63

*Unpaired t-test with Welch’s correction.

^†^Chi-square test.

Fifty-nine eyes of 52 patients had a follow-up of ≥5 years with a mean follow-up of 14.8 ± 9.4 years (range: 5–43 years). The mean age of these patients at the initial visit was 56.8 ± 16.1 (range: 24–78 years), and after excluding 24 pseudophakic eyes, the mean refractive error was −13.9 ± 5.5 D (range: −6.25 to −24 D). The mean axial length of the eyes was 30.1 ± 2.1 mm (range: 26.07–32.81 mm). When fundus photographs taken at the initial visit and at the last visit were compared with each other, none of the patients showed a newly developed CA during the follow-up period.

### Formation of CVs

**Figure 4:**
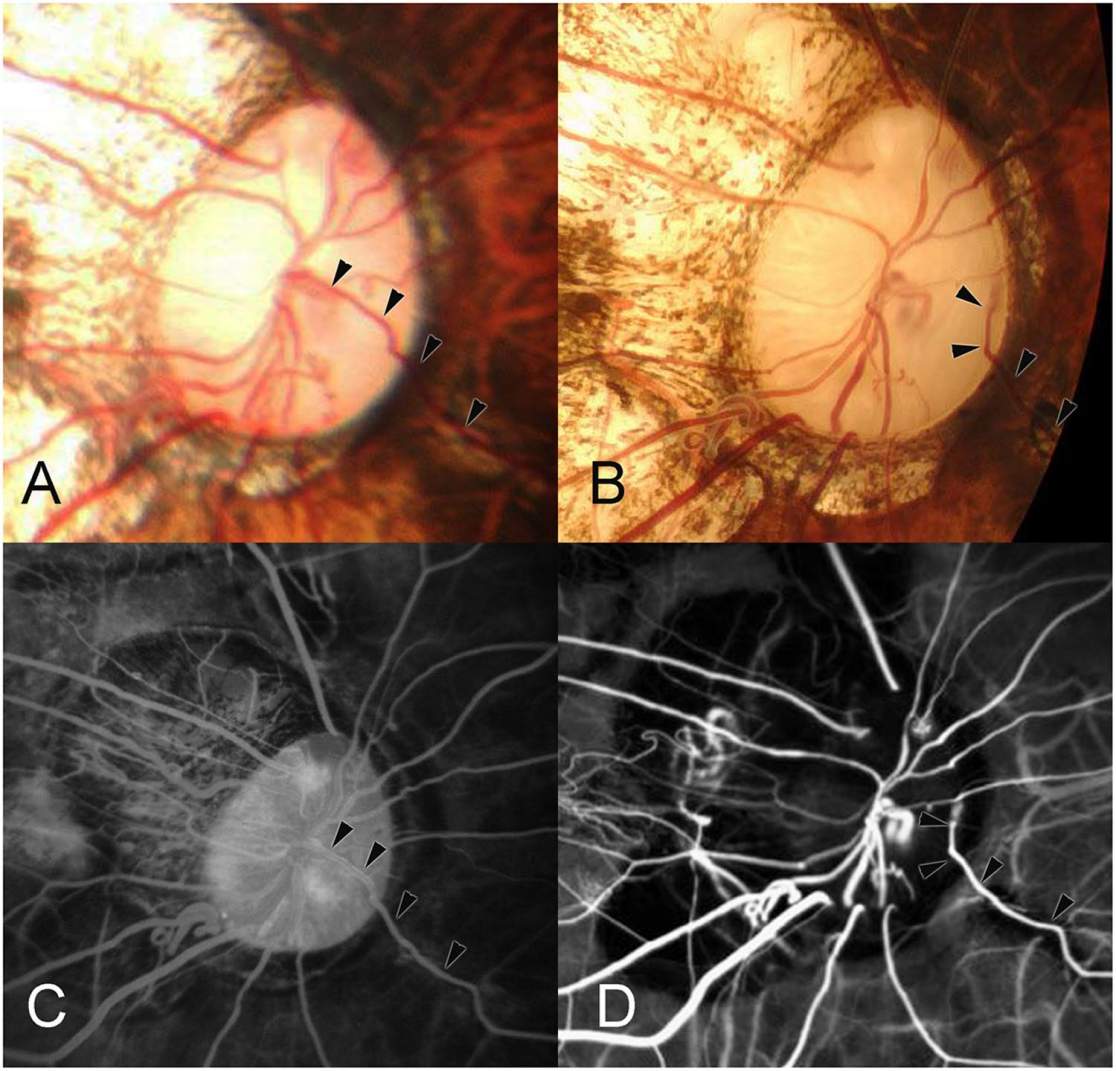
Development of a cilioretinal vein (CV). (**A**) Right optic nerve head of a 49-year-old woman with the retinal vein at 4 o’clock draining into the central vascular trunk (arrowheads). Axial length is 29.9 mm. (**B**) Eight years later, the same retinal vein (arrowheads) has changed its course and exits in a newly acquired (glaucoma-related) pit of the optic nerve head at the nasal optic disc border at 3 o’clock, separated from the central vascular trunk. Note: Far advanced glaucoma-like optic nerve damage with kinking of the retinal vessels close to the disc border. (**C**) Fluorescein angiogram at the initial visit shows that the inferonasal retinal vein being continuous with the central vascular trunk (arrowheads). (**D**) Indocyanine green angiogram 8 years later shows that the inferonasal retinal vein had changed its course and left the eye at the nasal optic disc border (arrowheads).

In one eye, a CV was newly detected at the photograph taken at the follow-up examination (Fig. 4). At baseline, the retinal vein drained into the trunk of the central retinal vein. At follow-up, this vein had changed its course and left the eye at the nasal optic disc border through a newly acquired pit of the optic nerve head, separated from the central vessel trunk (Fig. 4). The acquired pit of the optic nerve head might have developed in relation to the far advanced glaucoma-like optic nerve damage.

**Figure 5:**
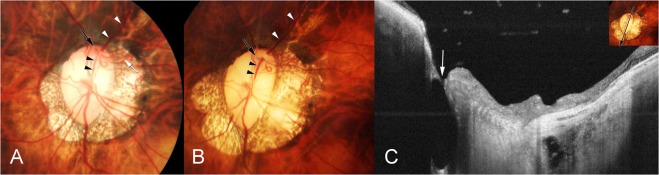
Dipping of a retinal vein into an acquired pit of the optic nerve head. (**A**) Left optic nerve head of the eye of a 49-year-old woman. The upper retinal vein (white arrowheads and black arrowheads) appears to be interrupted within the optic disc close to the disc border (black arrow). (**B**) Twelve years later, the length of the seemingly interruption of the retinal vein has enlarged, with the retinal vein dipping into a widening pit of the optic nerve head at the optic disc border at the 12 o’clock position. (**C**) Swept-source optical coherence tomogram showing the optic nerve head pit.

In another patient, a retinal vein dipped into a widening acquired pit of the optic nerve head close to the optic disc border so that the vessel contour appeared to be interrupted upon ophthalmoscopy (Fig. 5).

## Discussion

Earlier studies reported that CAs were found in 6% to 49.5% of the eyes in a general population^1–7^. In the present study, CAs were found in 7.3% of highly myopic eyes. However, this prevalence might not be fully representative, since only the patients who had undergone ICGA at our high myopia clinic were included into the study so that a selection bias might have occurred. A comparison between highly myopic eyes with and without CAs showed that the eyes with CAs had a significantly longer axial length and a significantly higher prevalence of glaucomatous optic neruopathy and macular atrophy. These results suggested that highly myopic eyes with CAs might represent more a more severe stage of myopia with a more marked damage to the macula and optic nerve. A possible relationship between CAs and glaucoma has been suggested by some authors^8^, although others did not find such an association^9–12^. Recently, Fang *et al*.^13^ reported that myopic macular atrophy (category 4 of myopic maculopathy) often developed from a myopic CNV. Due to a potential selection bias in our study, an influence of the CAs on blood circulation of the optic nerve head and the macula remains to be investigated in the future.

Earlier studies showed that CAs tended to supply mostly the temporal half of the retina and only rarely the whole retina^14,15^. In the non-myopic eyes included in these previous invstigations, the CAs were most often located in the temporal sector of the optic nerve head^1,3,16^. In contrast, in the highly myopic eyes included into the present study, the CAs were located predominantly in the superior optic disc sector (46%) followed by the temporal disc sector (37%). The reason of the difference in the predominant locations of the CAs between non-myopic eyes and highly myopic eyes has remained unclear so far. Due to the axial elongation, highly myopic eyes tend to develop acquired megalodiscs^17,18^. In eyes with megalodiscs, acquired optic disc pits can develop at the upper and lower pole of the optic disc in highly myopic eyes^19^. In some eyes, retinal tissue along with retinal vessels are embedded into these pits, and in rare instances, the retinal vessels may became connected with parapapillary vessels and may get disconnected from the central retinal vessel trunkAn acquired development of a clinically significant anastomosis between the central retinal artery and short posterior ciliary arteries in the laminar and prelaminar areas was reported in 16 year-old boy after the total resection of optic nerve glioma^20^. Another explanation would be in non-myopic eyes, that superior CAs might be missed funduscopically because too many overlying vessels might prevent the detection of the CAs. In the temporal sector of the optic disc as compared to the superior region, the number of vessels is markedly lower what may facilitate the detection of CAs.

By examining non-highly myopic eyes, Brown and Tasman^16^ assumed that CAs most commonly arose from the SPCAs and occasionally originated directly from choroidal vessels. In non-highly myopic eyes, however, the vessels the CAs derive from cannot well be visualized due to a relatively thick retina, choroid and sclera. In highly myopic eyes with a parapapillary gamma zone and delta zone and a thin retina, choroid and sclera, the ZHAC is usually located intrasclerally at the merging line of the optic nerve dura mater with the posterior sclera and is visible upon ICGA and optical coherence tomography (OCT)^6,7,21,22^. Taking advantage of these special anatomical conditions, we were able to examine the vessels from which the CAs arose. The results showed that 60% of the CAs arose directly from SPCAs while 40% of the CAs originated from the ZHAC. The results showed that the CAs located superiorly tended to originate from the SPCAs, and the ones located temporally tended to derive from the ZHAC. A PubMed search showed only one publication that reported a CA arising from the ZHAC. Ishida *et al*.^23^ examined the optic disc of highly myopic eyes by OCT angiography and reported on the presence of CAs arising directly from the ZHAC. The results of the present study revealed that the CAs originated relatively often from the ZHAC, especially the CAs located in the temporal region. The CAs arising the ZHAC may be called “ZHAC-associated CAs”.

Superior CAs originating directly from SPCAs tended to serve a large retinal area with some of the CAs spreading over almost one half of the posterior fundus (Supplementary Fig. 1). In a previous study, large CAs supplying more than a quarter of the retina were found in 0.6% of cases^24^. Lewis *et al*.^25^ reported on a 75-year-old woman whose CAs supplied her entire superior retinal hemisphere. The reason why CAs located superiorly tended to cover a larger area of the upper fundus may be that the arteries originated from larger SPCAs and not from the ZHAC.

The results showed that 69% of the ZHAC-associated CAs originated from outside of the optic disc border. Kim and coworkers reported that CAs located in the temporal region and primarily emerging within the optic disc moved during the process of myopia progression their emerging point to the parapapillary region outside of the optic disc^26^. One may discuss, that with the progression of axial myopia the peripapillary scleral flange further elongates so that the distance between the ZHAC and the optic disc border (defined by the peripapillary border tissue of Jacoby and Elschnig) increases^7^. In the case of CAs originating from the ZHAC, the increasing distance between the ZHAC and the disc border may be the reason for the movement of the emerging point of the CA outside of the optic disc in direction to the fovea. In our selected study population, the ZHAC-associated CAs emerged outside of the optic disc in 61% of the eyes, parallel to the presence of a delta zone in these eyes (Fig. 2). A parapapillary delta zone is the surrogate of an elongated peripapillary scleral flange.

The results also showed that the CVs ran parallel to the CAs in 14 of 95 eyes (15%). The prevalence of the CVs was considerably lower than the prevalence of the CAs. Correspondingly, only few studies examined CVs previously. The CVs have also been called “cilio-optic veins” or “optico-ciliary veins”, when they became ophthalmoscopically visible after a long-standing central or branch retinal vein occlusion^27–29^ or after a compression of the retrobulbar central retinal vein due to an optic nerve sheath meningioma. A PubMed search did not extract any publications that reported the presence of CVs without a central retinal vein occlusion or optic nerve sheath meningioma. None of the 14 eyes with CVs showed signs of a central retinal vein occlusion. It suggests that the CVs were present independently from these conditions in the highly myopic patients of our study population.

The finding that the CVs ran parallel to the CAs and that the CAs and the CVs appeared to penetrate the eye at approximately the same site suggests that the formation of CAs and CVs may be related to each other. In all of these 14 eyes, the CAs entered, and the CVs left, the eye in the superior region. In one of the 14 eyes, a posterior vortex vein was present close to the exit of the CV, although a clear connection between the CV and the posterior vortex vein could not be visualized upon ICGA. A posterior vortex vein was found near the penetrating site of the CV in about one-quarter of the highly myopic eyes^30^. A connection between the CV and the posterior vortex vein might be one possibility and may be investigated in future studies by ICGA.

A comparison of the patients with CAs between those with and without CVs showed that the eyes with both CAs and CVs had a significantly higher prevalence of glaucoma and a significantly lower prevalence of lacquer cracks than the eyes with CAs only. The meaning of this difference has remained elusive so far.

CAs have been reported to be present in eyes with congenital optic pits^31^. Congenital optic disc pits are characterized by a defect of the lamina cribrosa as determined by histological examinations^32^ and also by OCT^33^. We have also reported that acquired optic nerve head pits were common in highly myopic eyes^19^. In the present study, the presence of acquired optic pits at the penetrating site of CAs or CVs was not examined by OCT for all patients due to the retrospective nature of the study design. However, retinal and ciliary communication might occur for the arteries and veins through defects of the lamina cribrosa at the site of acquired optic pits due to pathologic myopia. One may discuss whether the extreme mechanical expansion of the papillary in highly myopic eyes could facilitate the development of new CAs in highly myopic eyes.

The strength of this study was to clarify the detailed features (including vessel origin) of CAs in patients with pathologic myopia. The features of co-existing CVs were also shown in detail. This study also has several limitations. First, due to the retrospective nature of the study design, not all of highly myopic patients who visited the High Myopia Clinic during the study period had FA or ICGA. Thus, there may be some potential bias for the selection of enrolled patients. Second, the presence of a CV was analyzed only in eyes with a CA. It is possible that CVs were present without co-existing CAs in some highly myopic eyes.

With those caveats in mind, the results of this study showed that CAs in eyes with pathologic myopia can be classified into two types according to their origin; SPCA-derived CAs and ZHAC-associated CAs. The former tended to emerge in the superior sector of the optic disc, and the latter emerged temporally from a temporally-dislocated ZHAC. CVs running in parallel with CAs were found in some eyes. An elongating peripapillary scleral flange in eyes with progressive axial myopia may increase the distance between the ZHAC and the disc margin and may lead to a movement of the emerging point of the ZHAC-associated CAs outside of the optic disc in direction to the fovea. The results showed that the retinal vascular system in the region of the optic nerve head was markedly altered in eyes with pathologic myopia. Future studies may address whether these vascular changes are related to the pathogenesis of high myopia-associated optic nerve damage.

## Methods

The medical records of highly myopic patients who were examined in the High Myopia Clinic of the Tokyo Medical and Dental University between January 2015 and December 2017 were reviewed. The patients showing CAs on fundus photographs, on fluorescein angiograms (FA) and on indocyanine green angiograms (ICGA) were selected. As controls, highly myopic patients who visited the High Myopia Clinic during the same period and did not have CAs on fundus photos, on FA or on ICGA were extracted from our database, and were used as a control. The procedures used in this study were approved by the Ethics Committee of Tokyo Medical and Dental University, and they conformed to the tenets of the Declaration of Helsinki. Written informed consent was obtained from the patients. High myopia was defined as a refractive error of <−8.0 diopters (D) or an axial length of ≥26.5 mm.

All participants underwent a comprehensive ocular examination including measurement of refractive error (spherical equivalent) and assessment of the axial length using laser interferometry (IOL master; Carl Zeiss Meditec, Oberkochen, Germany). Fundus photographs were taken using the TRC 50DX retinal camera (Topcon Medical Systems Co., Tokyo, Japan) or the VX-10i fundus camera (Kowa Co., Nagoya, Japan). FA and ICGA were performed applying the Topcon TRC 50 IA fundus camera or the HRA + OCT device (Spectralis®; Heidelberg Engineering, Heidelberg, Germany).

The diagnosis of CAs was based on the examination of the fundus photographs, FAs and ICGAs by two masked authors (TW, KOM) acting independently of each other. CAs were defined as retinal arteries emerging from the optic disc or from the parapapillary region and which were separated from retinal vessels directly or indirectly originating from the central retinal artery.

**Figure 6:**
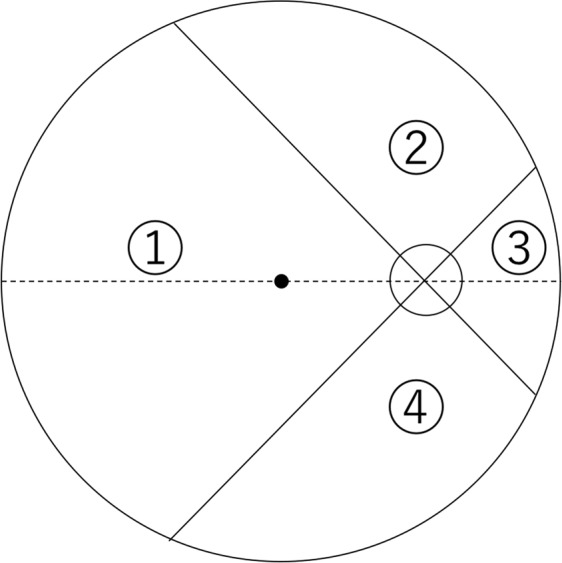
Division of the posterior pole of the eye to determine the location of the cilioretinal arteries (CVs). Zone 1, temporal; zone 2, superior; zone 3, nasal; and zone 4, inferior to the optic disc. Based on the line connecting the fovea and the optic disc center, the area including 45° upper from this line and 45° lower from this line was considered “temporal”, and then clockwise each 90° area was considered “superior”, “nasal”, and “lower”.

The number and the location of CAs were determined. The location of the CAs was classified as the quadrant where the CAs emerged into the retina (Fig. 6). The origin of the CAs either from the retrobulbar short posterior ciliary arteries (SPCAs) or the Zinn-Haller arterial circle (ZHAC) was assessed on the ICGA images taken during the arterial phase.

CVs were defined as veins which, on the fundus photographs, exited the eye separately from the central retinal vein trunk, and for which, on the ICGA images and on the FA images, no connecting vessels between them and the central retinal veins were detected. CVs were assessed by two masked authors (TW, KOM) independently of each other.

Finally, the fundus photographs of patients with a follow-up time of more than 5 years were examined to determine whether CAs and CVs were newly detected during the follow-up period.

Glaucomatous optic neuropathy was defined based on the appearance of the optic nerve head on the fundus photographs. The main criterion was an abnormal shape of the neuroretinal rim, including pronounced notches or an almost complete loss of neuroretinal rim mainly in the inferior or superior optic disc regions. The visibility of the retinal nerve fiber, the diameter of the retinal arteries, parapapillary alpha, beta, gamma or delta zones and intraocular pressure were not considered. Kinetic Goldmann visual field examinations the results of which were available at the time of the study were used to justify the diagnosis of GON in a subset of eyes.

Clinical features such as age, refractive error (spherical equivalent), axial length, subfoveal choroidal thickness, category of myopic maculopathy, the prevalence of the severe form of myopic maculopathy (≥diffuse atrophy), lacquer cracks, myopic CNV and glaucoma were compared between the patients with and without CAs, as well as between the patients with CAs and with or without co-existing CVs. The unpaired student t-test with Welch’s correction was used for the comparison of age, refractive error, axial length and subfoveal choroidal thickness. The Chi-square tests were used to compare the prevalence of lacquer cracks, myopic CNV, glaucoma, each lesion of myopic maculopathy and severe maculopathy. A *P* value less than 0.05 was considered statistically significant.

All data generated or analyzed during this study are included in this published article.

## Supplementary information

supplemental FIgure 1
