# Supplementary material for: Cilioretinal Arteries and Cilioretinal Veins in Eyes with Pathologic Myopia
Source: Sci Rep. 2019 Feb 21;9:2451. doi: 10.1038/s41598-019-38616-5 (PMC6384956; doi:10.1038/s41598-019-38616-5)
Supplement: Supplementary file 1 — supplemental FIgure 1 [file 41598_2019_38616_MOESM1_ESM.pdf]

Cilioretinal Arteries and Cilioretinal Veins in Eyes with Pathologic Myopia

Takashi Watanabe[1,2], MD, Kaori Kasahara[1], MD, Soh Futagami[1, 3], MD, Yuxin Fang[1], MD, Ran Du[1], MD, Muka Moriyama[1], MD, PhD, Kengo Uramoto[1], MD, Tae Yokoi[1], MD, Yuka Onishi[1], MD, Takeshi Yoshida[1], MD, PhD, Koju Kamoi[1], MD, PhD, Jost B. Jonas[4], MD, PhD, and Kyoko Ohno-Matsui[1], MD, PhD

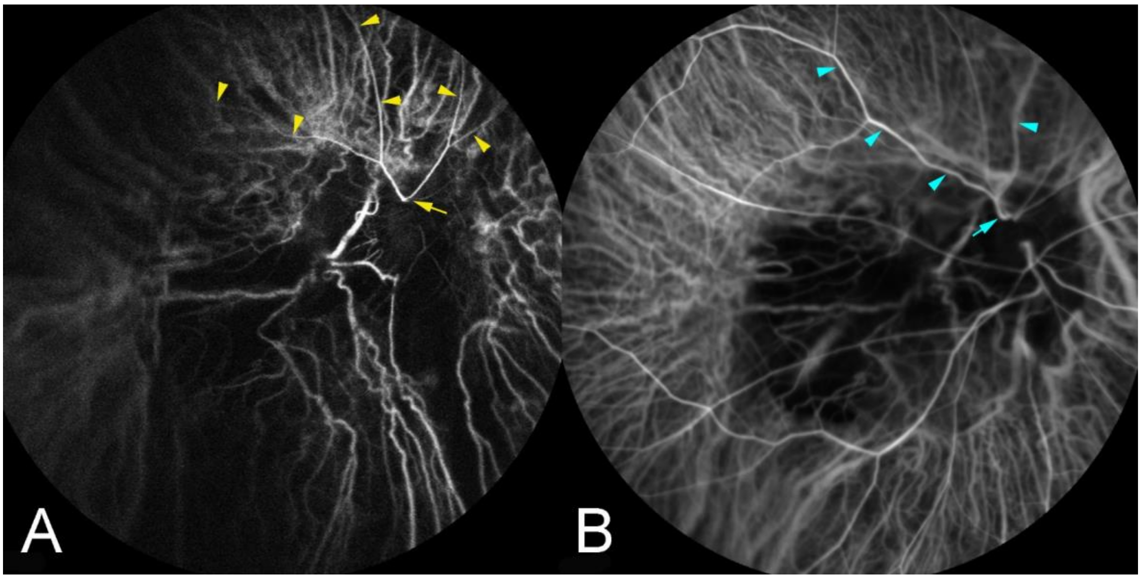

Supplementary Figure 1. Cilioretinal artery (CA) covering nearly one-half of the fundus.

Right fundus of the eye of a 78-year-old woman with axial length of 29.0 mm.

Supplementary Figure 1a. In the arterial phase of indocyanine green angiogram (ICGA), a large CA emerges at the point shown by the arrow and serves a large part of the upper half of the fundus (arrowheads).

Supplementary Figure 1b. In the venous phase of the ICGA, a large cilioretinal vein (arrowheads) runs parallel to the CA and exits the eye at the point shown by the arrow.
